# Supplementary material for: Complexome profiling of the Chlamydomonas psb28 mutant reveals TEF5 as an early PSII assembly factor
Source: Plant Cell. 2025 Mar 17;37(6):koaf055. doi: 10.1093/plcell/koaf055 (PMC12164590; doi:10.1093/plcell/koaf055)
Supplement: koaf055_Supplementary_Data [file koaf055_supplementary_data.zip › psb28_interactive.zip!/plots/intensity/qualityPlots/peptides/_Cre16.g674950.t1.2.html]

### Cre16.g674950.t1.2

Trivial name:   
SeqCoverage [median]: 12.90  
Mass: 60.17 kDa  
  
 

| Sequence | MissedCleavage | Mass [Da] | wtIntensity | muIntensity | Score |
| --- | --- | --- | --- | --- | --- |
| AELSSVGTPLTHER | 0 | 1495.76 | 508.32 | 1106.89 | 79.63 |
| AVSAPAKPTPK | 1 | 1065.62 | 413.35 | 545.09 | 45.08 |
| IGNDNFVEVLK | 0 | 1246.66 | 607.49 | 1443.30 | 108.67 |
| YDPGALVTAIGR | 0 | 1231.66 | 1199.82 | 955.62 | 77.38 |
|  |  |  | 2729.0 | 4050.9 |  |
